# Increased Pain Communication following Multiple Group Memberships Salience Leads to a Relative Reduction in Pain-Related Brain Activity

**DOI:** 10.1371/journal.pone.0163117

**Published:** 2016-09-22

**Authors:** Laura J. Ferris, Jolanda Jetten, Pascal Molenberghs, Brock Bastian, Fika Karnadewi

**Affiliations:** 1 University of Queensland, Brisbane, Australia; 2 School of Psychological Sciences and Monash Institute of Cognitive and Clinical Neurosciences, Monash University, Melbourne, Australia; 3 University of Melbourne, Melbourne, Australia; IRCCS Istituto Auxologico Italiano, ITALY

## Abstract

Pain is a fundamental human experience that triggers a range of social and psychological responses. In this study, we present behavioral and fMRI data to examine the effect of multiple group memberships salience on reported and neural indices of pain. We found that participants expressed higher levels of pain when more social group memberships were salient. This is consistent with the notion that pain itself motivates people to communicate their pain, and more so when multiple psychological resources are salient. In addition, fMRI results reveal an interesting twist: when participants increased their pain reporting as group memberships increased (from one group to four), there was a corresponding relative reduction in dorsal anterior cingulate cortex and anterior insula activation. These results provide evidence for an adaptive response to pain: the more people make use of the social resources at their disposal when experiencing pain, the less pain areas are activated.

## Introduction

Pain is a subjective sensory and emotional experience that contributes substantially to global disease burden [1–3]. Pain is private, subjective, and intrapersonal; but it is also experienced and expressed within a social context [4–8]. Communicating pain to others is a key step in securing aid and social support from others [9]. This makes it important to understand how social resources contribute to how pain is experienced and reported.

Social group memberships are one way to examine how social resources affect pain and its communication. Group memberships—and the social identity that we derive from them—can be considered valuable resources that people may draw upon in responding to pain. By self-categorizing as a member of a group, individuals derive *social* identity that dynamically informs their understanding of self relative to others [10]. The term ‘group’ is defined broadly, such that a person may self-categorize as a member of a social or demographic category (males, Americans), or other groups based on different parameters (scientists, cancer survivors; [11]). With relevance to pain, there is a growing body of work showing the role of group memberships in buffering and overcoming suffering [12–15]. It follows that membership in more groups should arm people to better respond to painful challenges, and if group memberships are resources, then more resources should flow from the salience of more group memberships. This notion builds on the idea of ‘the social cure’, whereby group memberships can deliver socioemotional and health benefits by building social resources [12].

However, it remains to be seen precisely how these resources affect pain communication. Combining neuroimaging with pain reports provides a way to understand pain on behavioral and neural levels [16, 17], and here we examine how these indices are affected by salient social group memberships. Does the salience of one’s group memberships *buffer* the pain experience and diminish the need to report pain (lower pain reporting), or alternatively, does it imply a supportive environment or ‘safe space’ to express and communicate our pain (elevated pain reporting)? We explore these opposing predictions in turn.

### Groups facilitate communication of pain

Group memberships are a scaffold for communication because they provide common language, goals, motivations, and ‘shared reality’ [18]. Although pain is a subjective, internal experience, pain can be conveyed to others through facial expression, non-verbal vocalization, pain behaviors, and language. The social milieu in which these processes occur plays a critical role in determining their nature and outcomes [4]. Expressing pain to our ingroup is functional: at a basic level, pain communication can aid the sufferer by limiting exposure to the nociceptive source and minimizing damage, as pain expressions and distress vocalizations provide valuable signals to conspecifics on potential risks, dangers, and ameliorative action required [9, 19–21]. Expressing pain can itself serve psychological functions—simply vocalizing pain improves pain tolerance, such as saying “ow” [22] or even swearing [23, 24]. Signaling pain is also a way to engender empathy and helping behaviors in others, because seeing others in pain elicits empathy and helping, particularly between ingroup members [25].

If expressing pain to those around us serves to enhance the provision of support, this strategy is more likely to serve as a functional response to pain when more social resources are available or perceived. On this basis, communicating pain in response to social groups is adaptive because it facilitates access to psychosocial resources, and may even carry its own inherent payoffs. In short, people may be more likely to express their pain the more social resources are salient.

### Buffering pain: What do groups offer?

An alternative line of reasoning shifts the focus to group memberships as a more direct buffer for pain. There is now a wealth of evidence demonstrating the positive impact of belonging to social groups on health and well-being, and the deleterious effects of social isolation [26, 27]. Social support is linked with reduced pain and lower analgesic consumption during childbirth [28, 29], better recovery from surgery [30, 31]; and improved pain adjustment in the context of chronic pain [32]. Reassurance from ingroup (versus outgroup) members during pain reduces physiological arousal measured by galvanic skin response [33].

It follows that if group memberships are an important resource during pain, the more of this resource one has, the better protected one is. Jones and Jetten [34] provided experimental evidence for the ‘more the merrier’ effect by varying the number of group memberships (1, 3 or 5) that were made salient to participants. Participants were asked to self-categorize in terms of either one, three or five social groups, and then to write about why each of the relevant groups was important to them. In conditions where more group memberships were salient, participants were able to endure physical pain from the cold pressor task for longer periods. Notably, when five groups were made salient participants were able to keep their hand in freezing water twice as long as participants for whom only one group membership was made salient. Aside from the tangible support that group membership provides, this suggests that the mere psychological availability and salience of social group membership acts as a resource for building resilience. However, questions remain. As Jetten and Jones’ [34] dependent measure involved resilience in the face of challenge, it is less clear how participants’ experience of pain itself was affected. Did participants *experience* less pain when more group memberships were made salient, or simply tolerate it for longer?

Brown, Sheffield [35] compared ratings of pain during the cold pressor task when a friend or stranger was present, versus pain alone. They also manipulated the type of contact: active support, passive support, or general interaction (i.e. involving unstructured talk with the other person present). While this study focused on a single interaction partner and not the salience of group membership, the results show that social support can reduce pain reports: compared to pain experienced alone, active and passive support conditions produced significantly lower pain reports than pain experienced alone. However, these effects were observed regardless of whether a friend or stranger was present. Furthermore, participants in the general interaction condition reported *more* pain than the active or passive support conditions, with mean pain levels no different to the alone condition. It is difficult to reconcile these findings with other experimental evidence of increased pain tolerance and reduced arousal when social resources are salient. Ultimately, the findings indicate that how pain is affected by others is not straightforward, and there is a need to consider further whether pain reports in the context of social groups are a function of painfulness, or an adaptive signal of responsive support-seeking.

### Measuring pain communication and pain-related brain activation

Pain is complex and multidimensional, and *pain* (a subjective unpleasant sensory and emotional experience) should be distinguished from *nociception* (the activation of pain receptors in the body; [3]). It is important to point out that pain self-reports are generally considered the gold standard in measuring and understanding a person’s pain [IASP, 1994/2016]; however, this is not without debate. Wager and Atlas [17] propose that pain self-report is insufficient to characterize the pain experience and the processes underlying pain (see also, [16, 36]).

The characterization of a diagnostic neurologic signature for pain has also been debated ([37, 38], see also, [39, 40]). A wide variety of brain areas are activated when experiencing pain, including the somatosensory cortex, cerebellum, thalamus, insula, cingulate cortex, as well as frontal and parietal areas [37]. In this study we were particularly interested in the dorsal anterior cingulate cortex (dACC) and anterior insula (AI) given that these regions across the neuroimaging literature are most consistently implicated when experiencing physical pain [41–43]. Insular regions are proposed to subserve representations of physiological states of the body as a foundation of interoception, including specific regions instantiating pain [44–47]. The dACC is posited to integrate pain, negative affect and cognitive control [48], and dACC activation also maps onto pain sensitivity [49]. This makes these regions appropriate candidates to examine the impact of salient group memberships on pain reports and brain activation patterns associated with pain. In an attempt to triangulate measurement, in our research we combine neuroimaging (i.e., the measurement of brain activity as an index of pain) with self-reports of pain.

### Overview of the research

In this study, we examined two ways in which group memberships can act as a psychological resource that affect responses to pain. First, focusing on the notion of pain as a signal of the need for support, we predicted that the more group memberships are made salient, the greater reporting of pain we would find, because more salient group memberships should elicit increased pain communication (H1; communication hypothesis). Alternatively, and rather straightforwardly, salient groups may buffer people from experiencing pain. This would lead to the prediction that the more group memberships are made salient, the less pain people will report (H2; buffering hypothesis). We also examined brain activation in dACC and AI in order to explore whether social group salience would impact pain reports and neural indices in the same way. We specifically looked at the change in these measures between the multiple-group and single-group conditions.

## Method

### Participants

Twenty participants (4 males) participated in the fMRI experiment (*M*_*age*_ *=* 22.45, *SD* = 1.99 years). All participants had normal or corrected-to-normal vision and cleared tests for MRI safety. We aimed to collect data from a sample of 20 and data collection ceased once this sample size was reached. All participants signed written informed consent and were reimbursed $30 for their participation. The study was approved by the University of Queensland Behavioural and Social Sciences Ethical Review Committee.

### Design, materials and procedure

The study involved a repeated-measures design. Pain and group salience were manipulated to create four conditions: three painful conditions (*multiple-group*; *single-group* and *multiple-traits*) and one non-painful condition (*control*). Participants presented individually for testing over two sessions scheduled no more than a week apart. In the first session, participants were briefed with study information, tested for fMRI safety, and signed written informed consent. Next they were asked to describe four social groups they identified with and that were important to them (for example, groups such as *university friends*, *church group*, *work friends*, or *yoga club*) along with four traits that described them well. These responses were then used to develop stimuli for use in the second session. For completeness and transparency, we describe and report the multiple-traits condition in our Method and Results sections. However, the condition is peripheral to our central hypotheses and is therefore not addressed in detail in the Results and Discussion.

**Fig 1:**
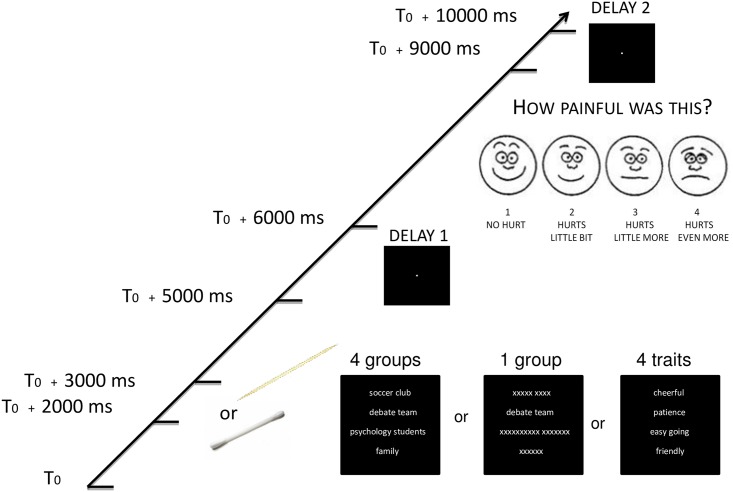
Schematic representation of an event during the fMRI experiment. At the start of the event, participants were presented with their four groups, one of their groups or their four traits for five seconds and during this time had to think about the words presented on the screen. After two seconds, participants received either painful (toothpick) or non-painful (Q-tip) stimulation. After six seconds, participants had three seconds to rate how painful the stimulation was. At the end of the event, a fixation dot appeared on the screen for 1 second.

In the second session, participants were briefed and invited to enter the fMRI scanner. The fMRI procedure consisted of five repeated functional runs (~8 minutes each), and a structural scan (~5 minutes) between the third and fourth run. At the beginning of each run, participants were presented with the instructions as a reminder. The instructions read: “You will see either: a) 1 group word, b) 4 group words or c) 4 traits presented on the screen during which painful or non-painful stimulation is applied at different intensity levels. During the stimulation try to think about the a) 1 group; b) 4 groups or c) 4 traits.” After the instructions at the beginning of each run, a white fixation dot was presented on a black screen for 7.5 seconds, followed by the event sequence. Each functional run consisted of 48 events consisting of the four different conditions (12 events per condition) which lasted 10 seconds per event (see Fig 1).

#### Group salience manipulation

In the *multiple-group* condition, at the start of each event, the four group-words from the first session were presented underneath each other for a total duration of 5 seconds (Fig 1). During these 5 seconds, participants were instructed to think about their four groups, which ensured that the relevant social resources would be salient during the pain manipulation. In the *single-group* condition a single group-word was presented for 5 seconds together with three lines of Xs which had the same number of characters as the missing words in the multiple-group condition. The Xs were used to match the visual stimulation in the single-group condition with that of the multiple-group condition to minimize differences attributable to variation in visual input. During this condition, participants were instructed to think about the one group. The particular group displayed was pseudo-randomly chosen for each event so that each of the four group-words was presented three times during each run. During the *multiple-traits* condition, the four trait words were presented on the screen for 5 seconds, and participants were instructed to think about these traits. In the non-painful condition, either four group words, one group word or four traits were presented (3 times each per run).

#### Pain manipulation

During the fMRI experiment, an experimenter was located next to the scanner wearing headphones. To implement the pain manipulation, the experimenter was instructed by two different 1-second beep tones to stimulate the participant’s finger for a duration of 1 second. In the three pain conditions, the experimenter applied painful pressure using a toothpick; while in the non-painful condition pressure was applied using a Q-tip. This procedure is similar to one used previously in the literature [50]. Crucially, the experimenter was blind to the presentation of group salience stimuli and unaware which groups condition (multiple-groups, single-group or multiple-traits) the participant was experiencing. Each beep tone was delivered to the experimenter 2 seconds after the start of the event; the stimulation lasted for 1 second so the participant was still thinking about the multiple groups, one group or multiple traits when experiencing the stimulation. After the group manipulation a 1-second fixation point appeared on the screen. Next, participants were given a 3-second response window to rate how painful the stimulation was by pressing one of four possible response buttons. These buttons corresponded with a 4-point rating scale based on the Wong Baker Pain Scale ([51]; see Fig 1), from 1 –‘no hurt’ to 4 –‘hurts even more’. Both pain rating responses and reaction times were recorded. At the end of the event, a fixation dot appeared for 1 second and then the next trial began. At the end of each run an 8-second fixation point was presented.

#### fMRI image acquisition

A 3-Tesla Siemens MRI scanner with 32-channel head volume coil was used to obtain the data. Functional images were acquired with the gradient echo planar imaging (EPI) with the following parameters: repetition time (TR) of 2.5 seconds, echo time (TE) of 36ms, flip angle (FA) of 90°. Thirty-six transversal slices with 64x64 voxels at 3mm^2^ in-plane resolution and a 10% gap in between the slices covered the whole brain. Whole brain images were generated every 2.5 seconds, and 202 images were acquired during each functional run. The first five images–during which no stimuli were presented—from each functional run were removed to allow for steady-state tissue magnetization. A three dimensional high resolution T1-weighted whole brain structural image was acquired after the third run for anatomical reference (TR = 1900, TE = 2.32ms, FA = 9°, 192 cube matrix, voxel size = 0.9 cubic mm, slice thickness = 0.9 mm).

#### fMRI analyses

We used SPM8 software (http://www.fil.ion.ucl.ac.uk/spm/) operated through Matlab (http://www.mathworks.com.au/products/matlab/) to analyze the data. To counter head movements all EPI images were realigned to the first scan of each run. The anatomical image was then coregistered to this mean functional image. To correct for variation in brain size and anatomy between participants, each structural scan was normalized to the MNI T1 standard template (Montreal Neuropsychological Institute) with a voxel size of 1x1x1mm using the segmentation procedure. The same segmentation parameters were then also used to normalize all the EPI images to the T1 template with a voxel size of 3x3x3mm. This process mathematically transformed each participant’s brain image to match the template so that any chosen brain region would refer to the same region across all participants. Before further analysis, all images were smoothed with an isotropic Gaussian kernel of 6mm.

As part of the first level of analysis, two general linear models were created for each participant. For each participant in each of the four conditions (i.e., no pain, multiple-group, single-group and multiple-traits), regions with significant Blood Oxygen Level Dependent (BOLD) changes in each voxel were identified using an event-related design time-locked to the time of the stimulation (i.e., model 1; 2 seconds after the start of the event) or at the start of the rating (i.e., model 2; 6 seconds after the start of the event).

In the second level of analysis contrast images for each condition across all participants were included in a factorial design. First a network was identified that was differentially activated for the painful minus non-painful conditions in model 1. We were particularly interested in the dorsal anterior cingulate cortex (dACC) and left and right insula (left AI and right AI) given the fact that these regions are most consistently associated with experiencing pain. Therefore, a region of interest (ROI) analysis was performed within the cingulate cortex and insula (anatomically defined by the WFU PickAtlas program: http://www.fmri.wfubmc.edu/cms/software). This analysis was thresholded at *p* < 0.001, and a voxel-level threshold with a familywise error rate (FWE) of *p* < .05 corrected for the size of the region of interest (ROI) was used to define significant activation. Subsequently, percentage signal change was extracted from the significant regions in this contrast for the three painful conditions for model 1 and 2 using the MarsBaR toolbox (http://marsbar.sourceforge.net/). We were particularly interested to see if people would communicate their pain more when thinking about multiple versus single group memberships and if this would lead to a relative reduction in dACC and AI activity. If increased pain reporting in the multiple-groups condition is more effective than in the single-group condition, the strongest relative reduction in activation would be present in model 2 (i.e., at the time of pain reporting).

## Results

### Behavioral results

#### Pain rating

**Fig 2:**
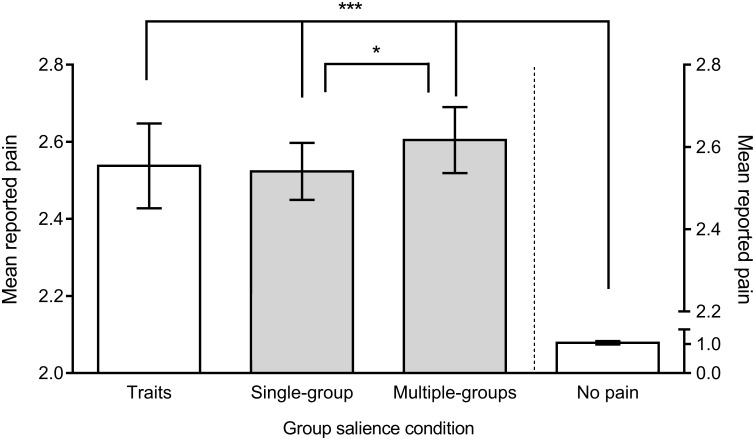
Mean pain ratings for the four conditions (no-pain control on the right *y*-axis). Higher scores indicate more pain reporting. Error bars represent 95% confidence intervals. Pairwise comparisons are Bonferroni corrected. *** *p* < .001; * *p* = .024.

A one-way repeated measures ANOVA revealed a significant difference in pain rating between the four conditions, *F*(3, 57) = 672.22, *p* < 0.001, η_*G*_^*2*^ = 0.94 (Fig 2; [52]). As expected, during the no-pain condition, participants reported less pain (*M* = 1.04, *SE* = 0.03) than in the multiple-group (*M* = 2.62, *SE* = 0.04, p <.001), single-group (*M* = 2.54, *SE* = 0.03; *p* < 0.001) and multiple-traits (*M* = 2.55, *SE* = 0.05; *p* <.001) conditions. Crucially, participants reported feeling more pain in the multiple-group (*M* = 2.62, *SE* = 0.04) than in the single-group condition (*M* = 2.54, *SE* = 0.03; *p* = 0.024, 95% CI_diff_ = [.008, .144]), although the amount of painful stimulation was the same in both conditions. No other differences were significant.

#### Reaction time

A one-way repeated measures ANOVA revealed a significant difference in rating speed between the four conditions, *F*(3, 57) = 8.05, *p* < 0.001. During the no-pain (*M* = 841, *SE* = 53) condition, participants responded faster than in the multiple-group (*M* = 947, *SE* = 47; *p* = 0.04) and multiple-traits (*M* = 959, *SE* = 49; *p* = 0.04) condition but not faster than in the single-group (*M* = 923, *SE* = 50; *p* = .14) condition. No other differences were significant.

### fMRI Results

#### Painful minus non-painful stimulation

**Fig 3:**
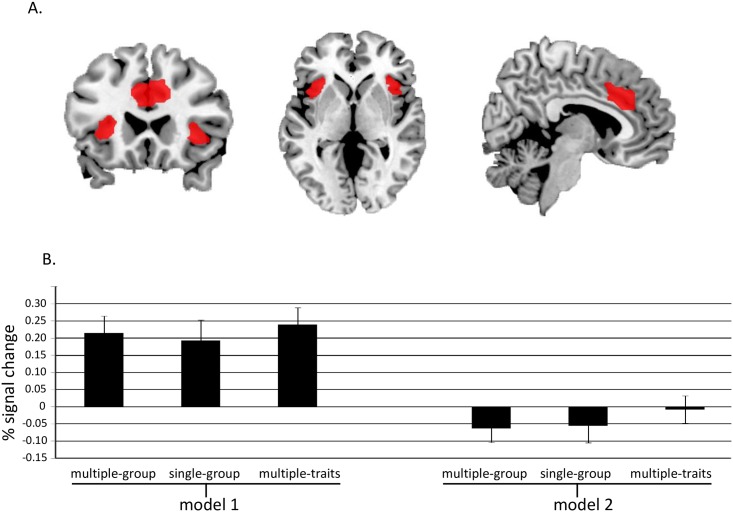
fMRI results and mean % signal change across conditions. (A) Significant brain activation for painful minus non-painful stimulation in left and right insula and dorsal anterior cingulate cortex displayed on a ch2better template using MRIcron software. (B) Mean % signal change for activations displayed in A for the three painful stimulation conditions at the time of stimulation (i.e., model 1) and at the time of the rating (i.e., model 2). Note: The baseline for model 1 and 2 is different (because they represent two different models) and therefore results between model 1 and 2 should not be directly compared against each other.

Significantly more activation was found in dACC (6, 20, 46; *k* = 93; *Z* = 4.81; *p* = .001), left (-33, 20, 7; *k* = 53; *Z* = 4.01; *p* = .028) and right AI (33, 17, 7; *k* = 25; *Z* = 3.88; *p* = .042; Fig 3). The % signal was then extracted from these regions combined for the three pain conditions, for model 1 and model 2. A repeated measures ANOVA revealed no difference in activation between the multiple-group (*M* = 0.21, *SE* = 0.05), single-group (*M* = 0.19, *SE* = 0.06) and multiple-traits (*M* = 0.24, *SE* = 0.05) condition for model 1, *F*(2, 38) = 0.98, p = 0.381. A similar repeated measures ANOVA revealed no difference in activation between the multiple-group (*M* = -0.06, *SE* = 0.04), single-group (*M* = -0.06, *SE* = 0.05) and multiple-traits (*M* = -0.01, *SE* = 0.04) condition for model 2, *F*(2, 38) = 1.53, *p* = 0.230. This indicates the pain activation was similar across the three conditions at the time of stimulation and rating, as might be expected given that the amount of painful stimulation was similar across the three conditions.

**Fig 4:**
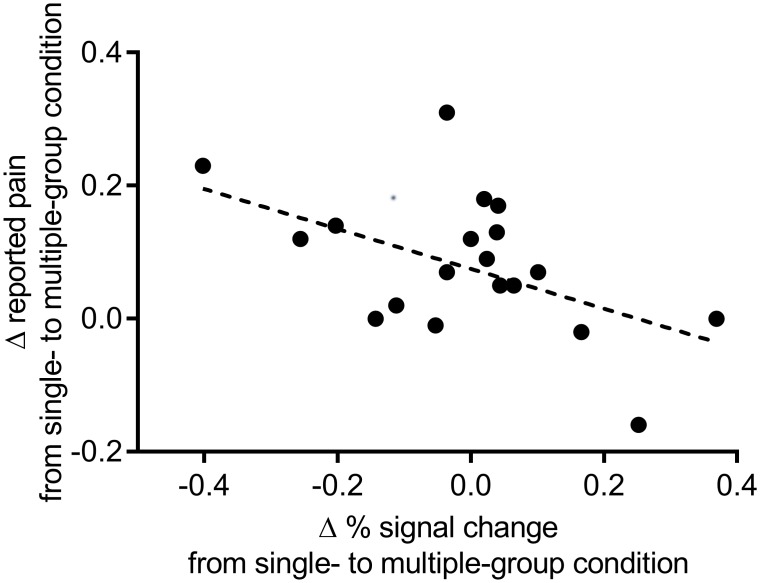
Scatterplot of differences in reported pain and % signal change (model 2) from single- to multiple-group condition. A relative increase in pain reporting as salient group memberships increased was associated with a corresponding relative reduction in dACC and AI activation.

More relevant is the question of whether increased pain reporting in the multiple- versus single-group condition leads to a relative reduction in dACC and AI activation. To investigate this, the difference in pain rating for the multiple-group minus the single-group condition was correlated with the difference in % signal change for the multiple-group minus the single-group condition (both for model 1 and model 2). A one-way Pearson correlation revealed a marginal negative correlation for model 1, (*r*(19) = -.35, *p* = .068) and a significant negative correlation for model 2 (*r*(19) = -.49, *p* = .014, *r*^2^ = .24; Fig 4). This shows that the more participants shared their pain in the multiple-group versus the single-group condition, the less activation was detected in dACC and AI in the multiple-group versus the single-group condition.

## Discussion

Pain is complex, private, and subjective, and the impact of social resources on how pain is experienced and communicated is not yet fully understood. In this study, we manipulated how many group memberships were salient to examine the effect of multiple group memberships on reported and neural indices of pain. We examined evidence for two competing hypotheses: first, that experiencing pain in the context of salient group memberships would lead to greater reporting of pain (H1; communication hypothesis). Second, that the salience of social groups that one belongs to would buffer people from experiencing pain, so that the more group memberships are made salient, the less pain people would report (H2; buffering hypothesis). We also examined brain activation in regions implicated in the experience of pain to explore whether the salience of a varying number of group memberships would impact pain reports and neural indices in the same way.

Behavioral findings were supportive of the communication hypothesis. Rather than a direct reduction in pain reports as a function of group membership salience, behavioral data showed that participants reported increasing levels of pain the more their group memberships were made salient. Specifically, participants reported more pain in the multiple-groups condition compared to the single-group condition. However the number of group memberships alone did not affect dACC and AI activation, which is consistent with the fact that the painful stimulus was the same across pain conditions. Instead, we found that ramping up pain reports as group memberships increased was associated with a corresponding relative reduction in dACC and AI activation. In essence, communicating pain by increasing pain reports in response to changes in social group resources was associated with a greater reduction in these neural indices of pain.

This finding is interesting and shows for the first time that being aware of social resources associated with group memberships enhances the extent to which pain is reported. It also demonstrates that making use of these social resources by increasing pain communication links with a corresponding relative reduction in brain activation in regions associated with pain. This adds to the literature on the role of group memberships as a psychological resource, particularly multiple group memberships [12, 53]. Social group memberships offer belonging, meaning, purpose, and even ‘existential security’ [10, 54–56]. This helps to explain why group memberships can provide particularly important social resources in times of adversity.

The present study’s findings are also consistent with other work highlighting the psychosocial utility of communicating pain. There are a range of barriers to effectively communicating pain to others [17], and pain is routinely underestimated by medical practitioners, parents, carers, and others [8, 57, 58]. However, communication of pain, particularly in the context of ingroups, enhances the likelihood that empathy is aroused and social support is provided [4, 9, 59]. The present study points to social group memberships as eliciting pain communication, and that even when invoked distally, the mere psychological availability and salience of social groups membership acts as a resource.

Making use of changes in these resources (i.e. by communicating pain accordingly) appears to impact the pain experience itself, based on brain activation data. The current findings therefore suggest that communicating pain in response to changes in the number of salient group memberships is a particularly functional and adaptive response to the subjective pain experience. Communicating pain is a way to secure the psychosocial resources that ingroup members can provide (see for example, [33]). However, the present study also provides insight into the potential emergence or maintenance of maladaptive pain responses, such as pain catastrophizing [60–62]. While there may be immediate benefits for communicating pain when there are more salient social resources, over time these social resources may become tapped, or unilateral signals may be misunderstood. This can lead to a mismatch between pain communication and responder support which can result in suboptimal care experiences and poorer outcomes [4, 62–64].

Critically, this study is the first to report a divergence in pain reports and pain-related brain responses and to this extent provides insight into the possibility that these indices of pain are not always in lockstep. Physiological and self-report pain measures might not always overlap and this has implications for how we measure and conceptualize pain [65]. The present findings show the informative value of measuring pain using different techniques, as both contribute to the scientific understanding of pain.

The present study also has limitations, such as the constraints on ecological validity inherent to research conducted within fMRI settings. In an effort to address this, we aimed to make the group membership stimuli as relevant and applicable to our participants as possible, by asking participants to nominate the particular groups that were important to them. We also took methodological steps to exclude distraction or inattention as possible explanations for the differences between conditions by ensuring stimuli were visually equivalent. One could argue that even if the visual stimuli are the same, participants might experience more distraction in the multiple-group condition vs. the single-group condition given that the task requires thinking about four groups instead of just one. However, if general distraction were at play, one would expect people to report *less* pain in the multiple-groups condition (because they are more distracted). Instead we find the opposite: people report more pain in the multiple-group condition vs. the single-group condition. We also found no differences in reaction times between the pain conditions. Therefore, it is unlikely that basic distraction could explain the pattern of findings in the present study.

In conclusion, by manipulating the number of salient group memberships that people belong to, we found that participants increased their pain reports when multiple group memberships were salient. However, when participants increased their pain reporting in response to the number of group memberships, there was a corresponding relative reduction in activation in brain regions associated with pain (dACC and AI). These findings point to an adaptive response to pain and suggest that group memberships act as psychological resources that can be brought into play during painful experiences. The more people make use of the social resources they have at their disposal when experiencing pain, the less pain areas are activated.
